# Dynamics in interprofessional learning: a focussed ethnographic study in a student-run dental clinic

**DOI:** 10.1186/s12909-025-08383-1

**Published:** 2025-12-19

**Authors:** Maria J. Kersbergen, Elske Hissink, Nico H. J. Creugers, Miranda G. H. Laurant, C. R. M. G. Lia Fluit, Wietske Kuijer-Siebelink

**Affiliations:** 1 Department of Dentistry, Radboud university medical center, PO Box 6960, Nijmegen, 6503 GL The Netherlands; 2 School of Health Studies, Research group Organisation of Healthcare and Services, HAN University of Applied Sciences, Nijmegen, The Netherlands; 3 Senior Lecturer in the Dental Hygiene programme, School of Allied Health, HAN University of Applied Sciences, Nijmegen, The Netherlands; 4 Radboudumc Health Academy, Department of Research on Learning and Education, Radboud university medical center, Nijmegen, The Netherlands; 5 Department of Oral Function and Prosthetic Dentistry at College of Dental Science, Radboud university medical center, Nijmegen, The Netherlands; 6 Department Radboudumc Health Academy, Innovative and person-centred learning and working in health care, Radboud university medical center, Nijmegen, The Netherlands; 7 Department Radboudumc Health Academy, Education and learning for interprofessional and transmural collaboration, Radboud university medical center, Nijmegen, The Netherlands; 8 Responsive Vocational and Professional Education, HAN University of Applied Sciences, School of education, Nijmegen, The Netherlands

**Keywords:** Interprofessional education, Interprofessional collaboration, Students, Healthcare, CHAT

## Abstract

**Background:**

Healthcare professionals, including oral healthcare professionals, increasingly collaborate across disciplines to address complex healthcare needs. New Dutch legislation is reshaping tasks, roles, and responsibilities, necessitating changes in collaboration between dental hygienists and dentists. Workplace-based interprofessional education (IPE) is crucial for preparing students for evolving practice. Research on IPE mechanisms in oral healthcare is scarce. This ethnographic study explores mechanisms promoting and hindering workplace-based IPE, focusing on collaborative behaviour and interprofessional learning among students in a student-run dental clinic (SRDC).

**Method:**

We conducted a focused ethnography with third- and fourth-year Bachelor’s dental hygiene students and first-, second-, and third-year Master’s dental students in a SRDC. Data included 36 h of observations across 20 field observation days, involving 18 dental hygiene and 24 dental students. Additionally, we conducted 31 interviews with 13 dental hygiene and 16 dental students. The data analysis was iterative, with thematic and open coding categorised based on patterns for deeper understanding of the data. We categorised codes under the core competencies of the IPEC framework to identify behaviours in the SRDC. We categorised codes according to the components and sub-triangles of the Cultural-Historical Activity Theory (CHAT) model, exploring their influence on behaviours in the SRDC. The CHAT model was used to analyse interactions and tensions, focusing on the division of labour, rules, and community influence. A combination of these techniques facilitated both descriptive and explanatory data interpretation, with reflective sessions ensuring consensus within the research team.

**Results:**

Students demonstrated collaborative behaviours such as utilising expertise, interaction, shared leadership, and guidance. However, competencies in ‘Values and Ethics’ and ‘Teams and Teamwork’ were less emphasised. The CHAT model revealed factors influencing IPE, including student uncertainty, feedback hierarchies, role conflicts, programme requirements, expertise conflicts, task perspectives, supervisory roles, leadership, physical proximity, time constraints, and pre-existing student relationships.

**Conclusions:**

In a SRDC, clear role delineation, reciprocal supervision, and active knowledge exchange foster a productive learning environment, though core competencies develop unevenly. Role ambiguity and integrating from distinct curricula impact collaborative behaviour. Enhancing communication, continuous feedback, and role diversity can optimise collaboration and enhance IPE, improving teamwork and patient outcomes.

**Supplementary Information:**

The online version contains supplementary material available at 10.1186/s12909-025-08383-1.

## Introduction

In the evolving healthcare landscape, including oral healthcare, interprofessional collaboration is increasingly recognised as essential for addressing complex care needs [1, 2]. Meanwhile, workforce challenges, including an impending labour shortage, have driven task shifting and redistribution among dental professionals [3]. In this study, the term ‘task’ refers to various aspects of roles and responsibilities within clinical practice, including patient care, specific procedures, patient education, and organisational duties. This broad definition encompasses the diverse activities in which students collaborate and share responsibilities. In 2019, the Dutch Minister of Health, Welfare and Sport (VWS) introduced a five-year pilot programme that granted dental hygienists greater autonomy, enabling them to independently perform procedures previously reserved for dentists, rather than carrying out these tasks under the dentist’s instructions [4]. This shift demands more careful and effective collaboration between dentists and dental hygienists, necessitating educational adjustments to equip them for independent practice of certain procedures [4]. Inadequate collaboration negatively affects not only the quality of patient care but also the job satisfaction of healthcare providers and the efficiency of the healthcare system [5]. As the emphasis on collaborative practice in oral healthcare grows, it becomes increasingly essential to prepare future oral healthcare professionals for team-based care [6]. This preparation fosters both profession-specific competencies and collaborative skills [7]. Interprofessional education (IPE) is essential in this preparation, as it lays the foundation for future professionals to engage in effective team-based care [8]. 

Four key mechanisms that foster the development of collaborative competencies in education, as outlined in the widely used Interprofessional Education Collaborative (IPEC) framework [9], are : (1) feeling responsible, (2) feeling enthusiastic/excited, (3) feeling safe, and (4) feeling ready [10]. Literature also demonstrates that dynamic and complex clinical teaching environments, with power imbalances and hierarchical relationships and/or perceptions, can hinder effective communication and collaboration [11–13]. This emphasises the critical role of psychological safety in facilitating open dialogue, constructive feedback and positive IPE experiences [14]. Recent research in dental education highlights how team dynamics and power relations significantly shape students’ perceptions and outcomes within interprofessional settings [15]. 

Prior studies underscore the importance of authentic learning environments for effective IPE. Specifically, workplace-based interprofessional learning is suggested to provide a more enriching educational experience than traditional classroom settings, as it enhances key aspects of professionalism and collaboration [10, 16, 17]. While the interaction between the learning environment (context) and previously mentioned working mechanisms has been recognised[18, 19], the specific dynamics within workplace-based learning and the associated collaborative behaviours that influence effective IPE remain largely unexplored.

In response to recent national developments in oral healthcare and dental education, the Student-Run Dental Clinic (SRDC) has emerged as an integrated, workplace-based learning environment embedded in both the dental hygiene and dental curricula. It provides students with opportunities to engage in authentic interprofessional collaboration with real patients under guided supervision.

In the Netherlands, as in many other countries, dental curricula differ from medical programmes. Unlike medical students, who often train in real-world healthcare settings, dental students receive clinical training in-house within their educational institution [20–22]. This unique structure offers interprofessional learning opportunities, exemplified by initiatives such as an SRDC. In these clinics, students practise interprofessional care in a real patient setting assuming defined roles within a structured learning environment [23]. At the SRDC, interprofessional collaboration primarily occurs between dental hygiene students (bachelor level) and dental students (master level), with each contributing their profession-specific competencies and responsibilities to the team. A detailed description of the SRDC, including its educational processes, competency-based structure, and supervision [24], can be found in Box 1.

**Figure:**
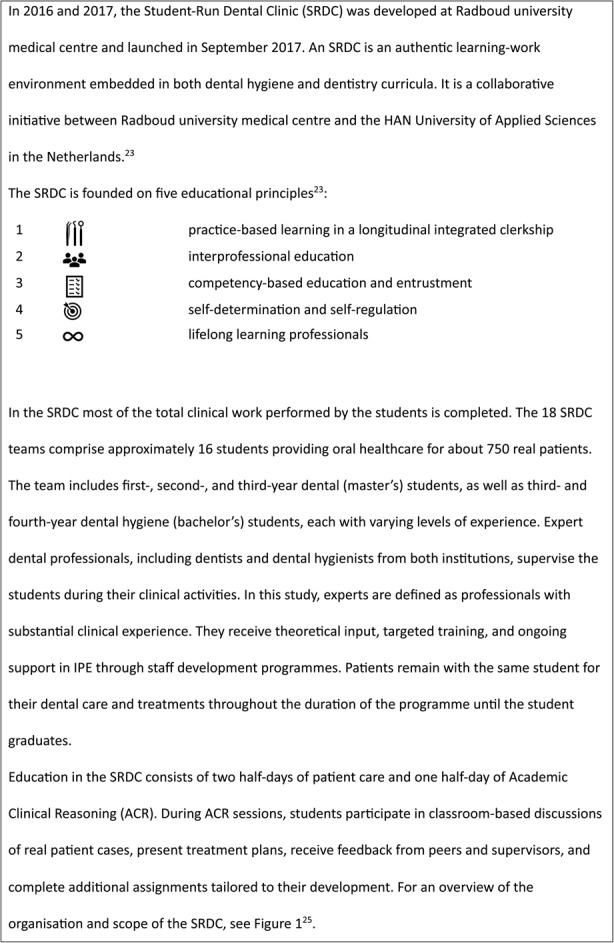
Box 1 The Student-Run Dental Clinic

**Fig. 1:**
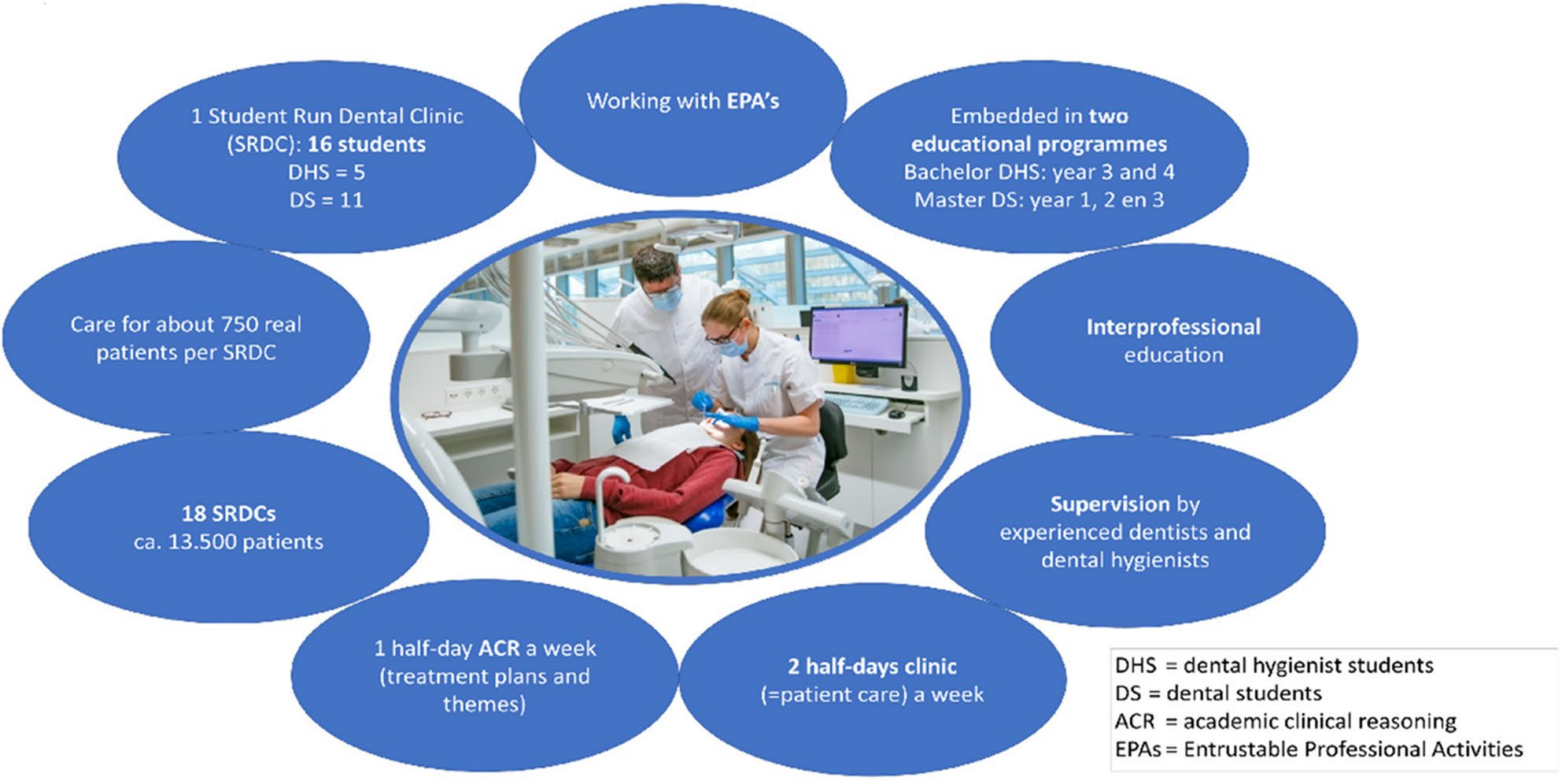
Characteristics of the Student-Run Dental Clinic. (adapted from Hissink et al., 2023)[25]

A previous study on SRDCs indicated generally positive attitudes towards interprofessional learning; however, differences exist between dental and dental hygiene students in their perceptions of collaborative learning and teamwork [26]. These differences may be influenced by regulatory changes that have reshaped professional roles, possibly affecting how students engage with interprofessional collaboration. Nevertheless, it remains unclear whether structured environments truly reflect the authentic workplace settings emphasised in prior research [16, 17]. Additionally, the ways in which workplace-based dynamics trigger key mechanisms for effective IPE require further exploration (Fig. 1).

Although oral healthcare has traditionally remained separate from general healthcare, the rising burden of oral diseases and their shared risk factors with other non-communicable diseases call for greater integration [2]. IPE in oral health not only strengthens collaboration between dental and dental hygiene students but also prepares them to work with a broader range of professionals. This supports the goals of the Quadruple Aim: improving population health, enhancing patient and provider experience, reducing costs, and promoting system sustainability [27]. 

Amid a changing landscape in oral healthcare, this ethnographic study examines the mechanisms that promote and hinder effective workplace-based IPE, focusing on collaborative behaviour and interprofessional learning in an SRDC. The study aims to answer the following research questions: (1) What collaborative behaviours can be observed in the SRDC? (2) What do students learn about collaboration through their collaborative behaviours in the SRDC? (3) What factors influence the collaborative behaviours in the SRDC?

## Methods

This qualitative study employed focused ethnography as the methodological approach. Ethnography is a well-established qualitative methodology used to explore the complexity of human interactions in natural settings, offering deep insights into social dynamics. Traditional ethnography typically involves long-term immersion, but focused ethnography is particularly suitable for investigating specific phenomena within a defined context when extended fieldwork is impractical [28, 29]. 

In this study, focused ethnography was chosen to examine the collaborative behaviour between dental hygiene and dental students within the SRDC environment. This approach is well-suited for exploring a clear and specific phenomenon—collaboration—while remaining time-efficient and contextually focused. By narrowing the scope to this particular aspect of the SRDC, focused ethnography enables an in-depth understanding of student interactions without the need for prolonged fieldwork.

### Reflexivity

The research team includes NC, an emeritus professor and founder of the SRDC; ML, a professor of applied sciences with expertise in the organisation of care and services; CF, a professor specialised in workplace learning; WK, a professor focusing on interprofessional learning and education; and EH, an educationalist and researcher engaged in the Dentistry programme who is familiar with the context. MK, the main researcher, is involved in the Dental Hygiene programme as a teacher. She conducted the observations and interviews, is familiar with the setting, and is trained in qualitative and observational research. A specific concern in focused ethnography is the researchers’ familiarity with the setting. A pitfall of this familiarity is that the researcher may take certain meaningful events for granted and cannot consider them ‘from a distance’, as the researcher shares implicit knowledge with the observed [28]. MK was aware of this ‘tacit knowledge’ and the obstacle it can cause in research. To mitigate this, she organised regular reflection sessions with the research team to critically discuss the observation reports and fieldnotes, ensuring that multiple perspectives were incorporated into the analysis. During these sessions, issues such as potential biases, alternative interpretations of the data, and the implications of the observations for the broader context were addressed. These discussions also enabled the team to critically examine how their diverse backgrounds and experiences shaped their perspectives, biases, and interpretations, particularly in relation to workplace and interprofessional learning in the SRDC. Reflexivity and intersubjectivity were further fostered through ongoing reflective discussions and reflective writing [28, 30]. 

These reflection sessions not only helped to mitigate the risk of bias but also enhanced the reflexivity of the research process. By incorporating multiple perspectives, they strengthened the credibility and transferability of the findings, making the insights more valuable for institutions aiming to establish similar interprofessional learning environments.

### Setting

The study was conducted within the SRDC in the Netherlands. Here, students from both the Dental Hygiene and Dentistry programmes across several educational years treat real patients and engage in collaborative learning and collaboration on Academic Clinical Reasoning (ACR). This designation reflects a broader range of responsibilities. ACR classroom-based sessions are designed to study and discuss real patient cases, where students discuss and prepare treatment plans or cases related to oral healthcare with one another. These sessions provide an opportunity for interprofessional learning by allowing students to work together, share insights, and plan care based on actual clinical scenarios. The clinic serves approximately 13,500 registered patients (see Box 1 for further details).

### Participants

Using purposive sampling, five of the 18 interprofessional teams were selected to ensure variation in supervision and scheduling. In the selection process, care was taken not to include multiple teams supervised by the same supervisor, to capture differences in supervisors’ professional backgrounds and teaching approaches. This variation was considered important, as the way supervision was provided could influence students’ interprofessional learning experiences. In addition, teams were chosen based on their scheduled clinic days, allowing observations to take place on different working days and with diverse team compositions. The teams were also selected based on their location (main clinic or satellite clinic) to capture contextual variation in clinical practice. This purposive selection was intended to enhance the richness and credibility of the data by including a range of supervisory practices, team dynamics, and clinical contexts.

All students in the selected teams were approached to be observed during clinical practice and during ACR, as well as in formal and informal communication related to patient care. The students of the five teams all received an information letter and signed a written informed consent form in advance.

Sample adequacy was guided by the principle of data saturation, defined here as the point at which no new codes, themes, or meanings emerged from the observations. Saturation was monitored through regular debriefings and comparative analysis by the research team during the data collection period. Saturation was considered reached when recurring patterns were observed and no new themes were identified, indicating that the data collected were sufficient to address the research questions within the study’s aims, context, and theoretical framework (including IPEC competencies and the CHAT model).

To prevent the students’ behaviour from being influenced by the presence of the researchers, participants were reassured in advance that their participation would not affect their assessments or relationships with fellow students. They were informed that the data would be handled anonymously and that their behaviour during the observations would not be used for academic evaluations. This was done to minimise potential bias and ensure the integrity of the research. Supervisors, dental assistants and visiting patients of the clinics received an information letter.

### Data collection

MK, the main researcher who is trained in qualitative research methods conducted data collection between March and July 2023, performing observations and interviews across five clinics during the ACR and clinic dayparts. The in-depth interviews took place consecutive to the observations and were scheduled in the same week as the observation. MK and EH systematically scrutinised transcripts and observation reports to iteratively refine the observation methodology. The first two weeks in March 2023 were used as an opportunity for pilot observations and to test and sharpen the topic list for observation and the interview guide with members of the research team (MK, EH, NC, ML, CF, WK). To enhance the quality of observation, fieldnotes, etc., regular consultations took place between MK and EH. The quality of the observations and interviews was monitored by writing reflective memos and frequent discussions with research team members (MK, EH, ML, CF, WK) who had extensive experience in qualitative research and ongoing training. This study combined interviews and observations to capture both perceptions and actual behaviour. The interviews provided insights into how participants experienced their interactions, while observations enriched and validated these self-reports. Both data sources were integrated during analysis to identify discrepancies and consistencies, offering a more comprehensive understanding of behaviour and interactions.

### Observations

Based on a previously conducted survey where we measured students’ attitudes [26] and results of a realist review on working mechanisms for IPE [10], we developed a topic list for the observation sessions (see Supplementary material 1). Various key events were observed non-participatory between March and July 2023, such as treatment plan presentations in ACR sessions and, during the clinical dayparts, patient intakes, treatment plan discussions with patients, combination appointments (where the patients were seen sequentially by different students). Reciprocal supervision between dental hygiene students and dental students during treatment procedures was also observed. Interactions in these events were observed, and fieldnotes were made according to three phases: (1) descriptive observations of the physical and social setting, general characteristics, followed by a focus on places, actors, and activities, (2) focused observations where the emphasis is on specific elements, themes, or aspects (cultural domains), closely aligned with the research questions; some domains were already delineated in the ‘topic list observation’, and (3) selective observations which begin once the observer is familiar with the setting and specific aspects. MK actively sought differences, contrasts, and paradoxes both between and within each student’s curriculum, aiming to uncover previously unnoticed significant aspects, as recommended in the literature [31, 32]. MK explained the observation process and addressed student questions at the outset. MK attended two observation training courses with EH.

### Interviews

The observed students were invited separately (individually) for a semi structured in-depth interview to explore attitudes, feelings and insight into their actions. The interview consisted of in-depth questions formulated by MK based on the observed event(s), with the aim of gaining a better understanding of the observed behaviour and communication. Possible questions to be asked in the in-depth interview were detailed in the interview guide (see Supplementary material 2). It should be noted that not all observations could be followed up with interviews due to constraints in time and the availability of students.

### Data analysis

#### Process of analysis

The data analysis followed an iterative process during observations and interviews. Fieldnotes and observation reports were shared and discussed with the research team to inform in-depth interviews and refining the observational topic list. A combination of qualitative coding methods was applied to break down the data into codes, with specific steps taken to analyse the data based on these codes, as recommended in the literature [33]. After reading transcripts and fieldnotes, initial coding was performed using Atlas.ti (version 23.3.4). The analysis was conducted in five phases, each with distinct objectives and methods, which are described below.

#### Phase 1: initial coding and thematic analysis

In the first phase, thematic and initial open coding was conducted based on IPE literature [9, 10]. Five transcripts were independently coded by two researchers (MK and EH) and subsequently discussed collaboratively. The purpose of this process was to enrich the analysis through reflection on different perspectives. Divergences in interpretation were used to deepen the understanding of the data and refine the preliminary coding framework, thereby contributing to analytic rigour and transparency. These discussions, sparked conversations within the research team, leading to refinement and application of additional codes across the dataset. This phase focused on identifying key themes related to collaboration in the SRDC, which were then used to inform the next stages of analysis.

#### Phase 2: refining the coding

In the second phase, we expanded on the initial coding by analysing all transcripts using both thematic and open codes [33]. The codes were grouped into categories by recognising patterns and similarities across the data. This analytical approach ensured a deeper understanding of the data as the analysis progressed, allowing us to identify recurring themes and establish connections between codes.

#### Phase 3: categorising codes using IPEC

##### Framework for analysis

To identify collaborative behaviours, we used a directed content analysis approach using the four interprofessional core competencies (Values and Ethics, Roles and Responsibilities, Communication and Teams and Teamwork) [9] as an analytical lens. Behaviours were considered ‘collaborative’ when they reflected interaction between students that contributed to shared patient care, mutual learning or role coordination. Examples included giving and receiving feedback, consulting one another on decisions, jointly managing a case, and reflecting on team dynamics.

To structure the analysis, we applied the Interprofessional Education Collaborative (IPEC) framework, which defines interprofessional competencies across these four core domains [9]. These competencies are essential for fostering collaboration in both educational and practice settings. In the third phase, we organised the data according to the IPEC domains to explore how these competencies developed in the SRDC and influenced collaborative behaviours, specifically addressing research questions 1 and 2. Findings were organised by domain, with behaviours linked to relevant (sub)competency and supported by illustrative quotations presented in Tables 1, 2, 3 and 4 in Supplementary material 3.

#### Phase 4: analysing tensions with cultural-historical activity theory

**Fig. 2:**
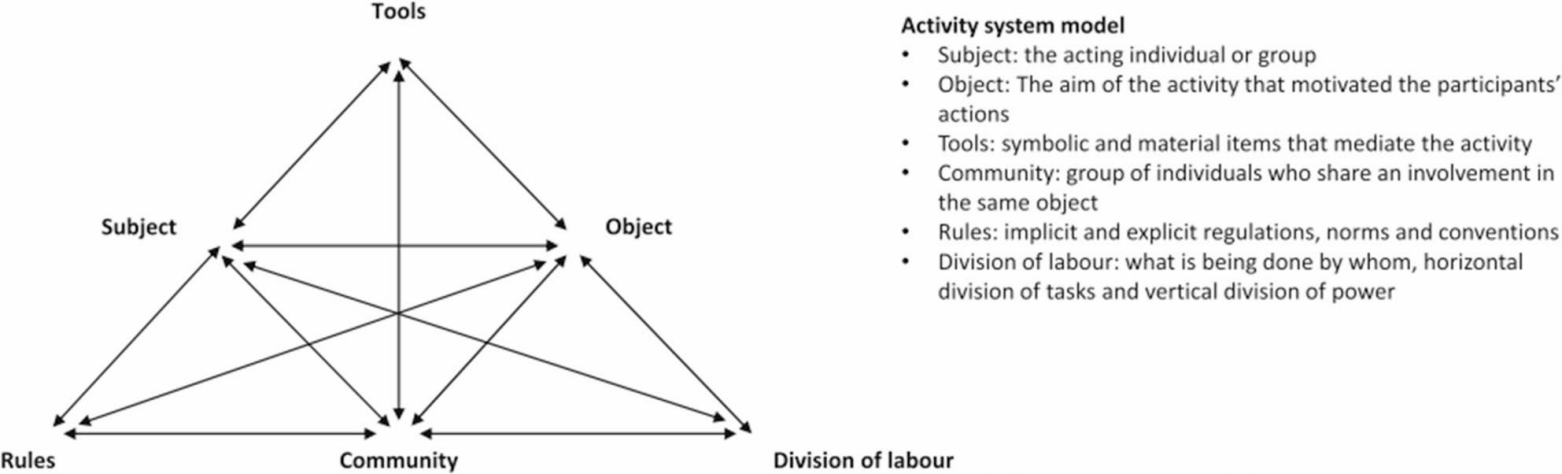
Activity system model. Notes: Reproduced with permission from © BMC Medical Education [39]

To address the third research question, we applied the Activity System Model from Cultural-Historical Activity Theory (CHAT) [34] to categorise the codes. CHAT (Fig. 2) provided a framework for understanding learning dynamics and identifying tensions in complex interprofessional learning environments [35–37]. This model enables visibility into relationships between components, showing how changes in one element can affect others [38, 39]. Understanding these interdependencies helps optimise interprofessional education for collaborative practice.

In our study, the SRDC context is viewed as a single activity system. By considering SRDC as such, we were able to analyse the dynamic interactions, shared responsibilities, and challenges that arise in interprofessional collaboration, ultimately enhancing the effectiveness of collaborative practice training. CHAT provided an analytical tool for examining interactions and tensions within the SRDC environment. We analysed tensions between elements such as the division of labour (tasks), organisational rules, and the community’s influence on workflow. The codes were categorised under the components and sub-triangles in the CHAT model. This approach provided deeper insight into the factors influencing collaborative behaviours. Supporting quotations for this stage are summarised in Table 5 in Supplementary material 4.

#### Phase 5: final refinement and synthesis

In the final phase, we refined our approach to achieve both a descriptive and explanatory interpretation of the data. By integrating insights from both frameworks, we achieved a comprehensive understanding of collaboration in the SRDC, which helped address all three research questions. Throughout the process, reflective sessions with the research team ensured consensus on the phenomena presented in the results section, ensuring the rigour and validity of our analysis.

To visualise the analytic process and the transition from inductive to theory-informed analysis, we present an overview of the five analytic phases in Table 1.

**Table 1:** Overview of the five analytic phases and their analytic focus

Phase	Description	Analytic focus
Phase 1	Initial coding and thematic analysis	Open/thematic coding based on literature and data
Phase 2	Refining the coding	Refinement and categorisation of patterns
Phase 3	Categorising with IPEC	Structuring codes using the IPEC framework
Phase 4	Applying CHAT framework	Analysing tensions through CHAT-informed categorisation
Phase 5	Final refinement and synthesis	Synthesizing descriptive and explanatory insights

## Results

Out of 20 field observation days, a total of 36 hours of observations were analysed, involving 18 dental hygiene students and 24 dental students. Additionally, 31 interviews were conducted with 13 dental hygiene students and 16 dental students. Some students were observed several times with a range from once to 7 times and were interviewed either once or twice. During observation sessions, multiple students were observed simultaneously while collaborating around the patient in the clinic or during ACR classroom activities. The dental hygiene and dental students followed a full-time educational programme. The age range for 3rd and 4th-year bachelor dental hygiene students was 19–21 years, and for 1 st, 2nd, and 3rd-year master dental students, it was 22–24 years Table 2.

**Table 2:** Demographic characteristics of participants

Students	Female	Male	Observations	Interviews
3rd Year bachelor dental hygiene	11		24	9
4th year bachelor dental hygiene	7		10	6
1 st year master dentistry	9	2	19	8
2nd year master dentistry	2	3	8	2
3rd year master dentistry	6	2	8	6
Total	35	7	69	31

### Observed collaborative behaviour and learning effects

To understand how collaboration unfolds in the SRDC, we first examined the types of collaborative behaviours students exhibit and what they learn from these interactions (research questions 1 and 2). Based on our analysis of the core competencies, we identified five types of collaborative behaviours, which are described below. The observed behaviours were closely aligned with the (sub) competencies of Roles and Responsibilities and Communication, while Values and Ethics and Teams and Teamwork were observed less frequently. The tables referenced in this section present quotations that are linked to the (sub) competencies [9]. 

#### Utilising each other’s expertise

Observations during ACR sessions revealed a distinct division of presentation roles, with students primarily discussing their own areas of expertise. In cases involving complex oral health issues, dental students assumed a more prominent role (Table 3-C1a, C1b). Collaborating to prepare treatment plans enhanced students’ understanding of each other’s roles (Table 2-RR1a) and facilitated knowledge exchange, particularly regarding alternative solutions. Dental students recognised the contributions of their dental hygiene peers, acknowledging the importance of their involvement for optimal patient care (Table 2-RR3a, RR4a). In the clinic, students engaged in discussions on accurate patient data collection methods, exchanging knowledge to improve care quality (Table 1-VE10a, Table 2-RR4b). They emphasised that collaboration herein and subsequent discussions on treatment plans underscored the significance of communicating their expertise. A dental hygiene student noted that this collaborative effort fostered a positive environment and encouraged a focus on shared goals (Table 3-C1c, C4a), a sentiment further supported by students’ reflections on teamwork. This was illustrated by one dental hygiene student who described how collaboration led to more comprehensive and integrated treatment planning:

“*Yes*,* by working together we end up having a better treatment plan than if you were to do it separately*…” (Table 4-TT6a).

#### Engagement and interaction

During treatment plan presentations in the ACR setting, group members exhibited attentiveness and respect towards the presenters (Table 3-C5a), with notable engagement and interaction, including active question-and-answer sessions (Table 3-C5b). Faculty encouraged this engagement by inviting student inquiries, fostering collegiality (Table 3-C6b). Interviews indicated that students valued active participation from their peers, which facilitated collaborative thinking and constructive feedback on treatment plans from both tutors and fellow students (Table 1-VE5a; Table 2-RR2a; Table 3-C5c; Table 4-TT3a).

The dynamics of interaction were influenced by group composition, with higher engagement noted when dental hygiene and dental students were evenly distributed, though the typical ratio was one-third dental hygiene students to two-thirds dental students (Table 1-VE10b). Participation varied by topic; discussions that became overly technical often saw a decline in dental hygiene student involvement (Table 3-C3a). Nevertheless, one dental hygiene student demonstrated strong commitment to the joint treatment plan despite the proposed solution being outside her area of expertise (Table 2-RR1b).

Collaboration was also evident in the clinic during the patient intake process, where students alternated roles in patient data collection based on their expertise. This was illustrated by one dental hygiene student who described how tasks were shared and responsibilities alternated:

*“We collected the data together. We gather the treatment plan data beforehand. She had taken the light photos*,* made the scan*,* and taken the X-rays*,* which were smaller tasks. I completed the periodontal status myself*,* but she was present and helped with that. She also heard the advice and instructions I provided during that session*,* and vice versa.”* (Table 2-RR1c).

In the absence of a designated role, they often acted as assistants and occasionally shifted roles to gain firsthand experience (Table 1-VE7a, VE11a).

A specific instance involved a dental student and a dental hygiene student coordinating a ‘combination appointment’ for a patient. The dental student conducted a periodic oral examination before the patient proceeded to the dental hygiene student. When asked why a dental hygiene student did not conduct the periodic oral examination, it was explained that the patient preferred consistency in practitioners throughout the treatment, with a dental student serving as the case manager (Table 3-C1d, C1e).

Differences in engagement levels were observed, with some dental hygiene students waiting in their respective units for patients while others chose to observe the examination. One student commented on the perceived flaws in the handover process from the dental student (Table 3-C1f).

#### Shared leadership

Shared leadership was observed among presenters, with students collaborating effectively during group discussions and utilising non-verbal communication, such as eye contact with faculty for affirmative nods, during presentations (Table 4-TT4a, TT4b). One dental hygiene student noted that collaboration fostered an understanding of shared leadership roles, including taking the lead in drafting treatment plans. This was illustrated by the student’s reflection on how decisions were negotiated and responsibilities shared:

*“He had initially come up with different solutions for this patient*,* more towards surgery and things like that. So based on that*,* we were just able to spar well that yes*,* that one or the other conceived path is not really appropriate for this patient. So*,* I do feel that I also had direction in writing this treatment plan.” *(Table 4-TT4c).

In the clinic, the dental student frequently provided explanations during treatment plan discussions with patients, while the dental hygiene student actively contributed insights related to her expertise or responded to invitations from dental students for clarification (Table 2-RR1e; Table 3-C1g).

### Resolving difficult situations in interprofessional collaboration

In one case, initial dissatisfaction with the collaboration process was noted, but providing feedback helped address these issues. Observations indicated an unequal contribution to the treatment plan’s development, leading to ambiguity regarding whether both dental and dental hygiene students had seen the patient together prior to drafting the plan (Table 4-TT7a). The dental hygiene student reported her absence during the patient visit (Table 3-C6d), which a dental student confirmed was inconsistent with established guidelines (Table 3-C6e).

#### Providing guidance and coaching

Observations of peer supervision revealed that dental hygiene students frequently supervised and coached dental students in periodontal treatments, while dental students provided oversight during preparations and restorations. These interactions involved significant engagement, with students asking questions, providing feedback, and sharing knowledge about their skills (Table 2-RR1f, RR1g; Table 3-C6f, C6g). Both groups reported extensive learning from one another during treatment implementation. For instance, one dental student noted that receiving practical tips enhanced treatment delivery (Table 2-RR1h). Additionally, students found that teaching each other about their skills prompted critical thinking about treatment steps and consideration of alternative approaches. This process also involved providing guidance and coaching, as one student reflected on how explaining and observing prompted reflection and allowed them to give feedback:

*“You become aware of what you yourself normally do somewhat automatically anyway. So you do learn to think about the steps again. And when they ask something*,* you naturally have to think: well*,* why do you do it like that? Or I think that’s mainly the effect it has. Because normally*,* of course*,* you are working by yourself with an assistant next to you who follows*,* well*,* and in this way you are also coaching a little and watching and giving some tips.”* (Table 2-RR1i).

### Factors influencing collaborative behaviours

Next, we examined the various factors that shape and influence these collaborative behaviours (RQ3). By exploring the context and underlying conditions, we gained insight into what facilitates or hinders effective collaboration.

**Fig. 3:**
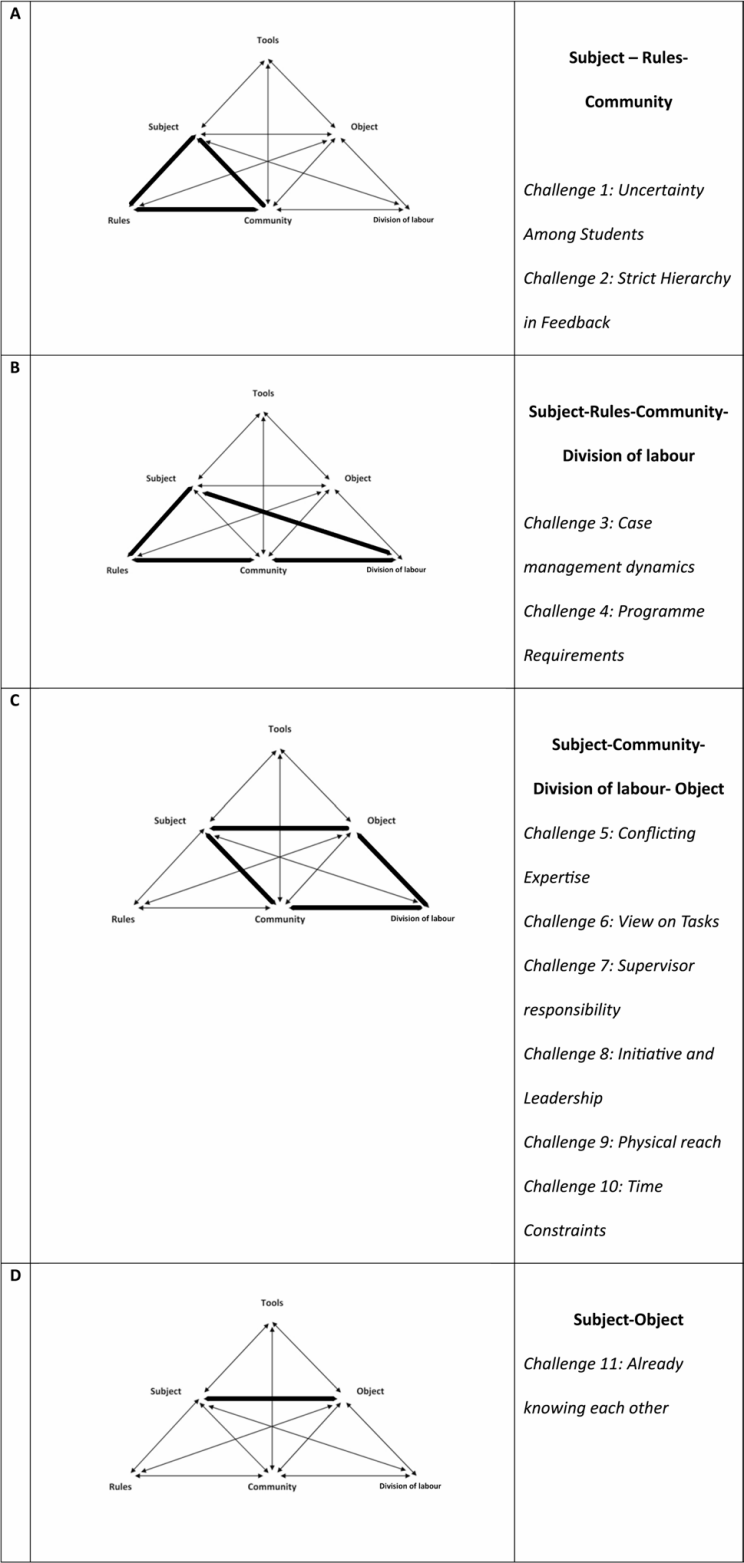
Challenges in collaborative behaviour and learning in four distinct sub-figures (A-D) of the CHAT- model

We identified eleven challenges which related to four distinct sub-figures (A-D) of the CHAT- model and the interactions between one or more activities within the CHAT-model (Fig. 3). The tables referenced in this section present quotations related to the challenges related to the distinct sub-figures of the CHAT-model.

### Subject-rules-community

#### Challenge 1: uncertainty among students

During treatment plan presentations, the dental hygiene student (subject) reported feeling unsure about their knowledge compared to dental students, which led to a fear of making mistakes. This uncertainty was evident at the start of participation in the SRDC, manifesting as self-doubt about contributing to the case discussions. The student illustrated how small contributions from peers gradually increased their confidence:

*“I noticed that in the third year I did struggle a bit more with that*,* also because I thought maybe I had a comment of oh*,* yeah*,* that’s irrelevant anyway… Then it occurred to me that somehow another X-ray had to be taken of that tooth. And I hadn’t mentioned that. But eventually that comment came through another student. So little things like that*,* and now I do dare to say it*,* I feel confident enough because of that.”* (Table 5 − 1).

#### Challenge 2: strict hierarchy in feedback

Younger dental students often hesitate to question senior peers, particularly early in their SRDC participation, due to the strict, hierarchical feedback from sixth-year students, who tend to dominate discussions. Both fourth-year dental students and dental hygiene students have noted this dynamic (Table 5-2a). In particular, dental hygiene students emphasised the importance of a more egalitarian approach, opposing the insistence that there is only one correct way to handle tasks (Table 5-2b).

One dental student illustrated how this hierarchical feedback affected their willingness to ask questions:

*“When I came into the SRDC in my fourth year*,* then we had some sixth years who pretty much said: this is wrong*,* so then*,* I thought*,* that’s how it came across to me*,* at least*,* I don’t know if that’s probably how they meant it*,* but that’s how it came across to me. So*,* I never dared to ask so many questions …”* (Table 5-2a).

### Subject-rules-community-division of labour

#### Challenge 3: case management dynamics

Both student groups reported that dental students consistently assume the role of case manager, an unwritten rule acknowledged by the students themselves. This expectation influences behaviour within the community and shapes the division of labour (Table 5 − 3). One dental hygiene student described this dynamic succinctly:

*“That’s dentistry always. The dental student is always the case manager and oral hygiene comes in as it were.”* (Table 5−3).

### Challenge 4: programme requirements

Collaboration and seeking assistance between dental hygiene students and dental students were partly driven by the programme requirements they both needed to fulfil for their training. For instance, dental students had to perform several periodontal treatments, while the dental hygiene students were responsible for preparing and restoring a few primary cavities. One student described how these requirements created opportunities for joint work:

*“Because I still needed those procedures*,* and she said*,* ‘Oh*,* I have an extra patient now. We can do them together then.”* (Tables 5 − 4).

### Subject-Community-Division of labour-Object

#### Challenge 5: conflicting expertise

The overlap in professional tasks between dental hygiene and dental students, as well as their perceptions of their own expertise and that of their peers, was evident both in direct patient care and in group discussions about the treatment plan. This influenced decisions regarding the division of labour. One dental hygiene student explained how differences in professional expertise shaped collaboration and role allocation:

*“And I also do see the patients sometimes for check-ups*,* but that’s not my main*,* treatment say so they are more likely to be able to draw out the patients’ problems and want to write a plan on that. So*,* they have that more often than we do. So that’s why dentistry says of oh*,* I want to write a plan with that patient*,* because I’ve seen this and this. Do you want to help with that?”* (Table 5–5).

#### Challenge 6: view on tasks

Tensions emerged regarding the division of labour and differing views on responsibilities, such as when a dental student commented on which patients’ dental hygiene students could or could not treat. The dental student’s perspective did not reflect the full scope of the dental hygienist’s expertise, instead limiting it solely to dental cleaning (Table 5-6). One interview illustrated this constrained view of responsibilities:

*“We do the other things*,* but they really handle the cleaning and maintenance. And then patients always come for follow-up*,* check-ups and cleanings at dental hygiene*,* and for the semi-annual check-ups they come to us. Interviewer: So that is not also done by dental hygiene? The dental student said: The semi-annual check-up*,* very rarely*,* they might do it once*,* but the next time it has to be a dental student again*,* but sometimes they do take over. …but difficult patients with prosthetics and things like that and frames are not handled by them for check-ups*,* so just the easier patients.”* (Table 5-6).

#### Challenge 7: supervisor responsibility

We observed that the supervisor played monitoring, initiating, and expert roles in the clinic during treatment implementation. Meanwhile, feedback was provided by supervisors from both programmes to the students, with supervisors aligning their approaches. In our observations, there was an instance where a dental supervisor chose not to provide guidance to a dental hygiene student due to disagreement with the evolving role of the dental hygienist. For example, one dental supervisor transferred the task of supervising preparation and restoration to a colleague, as he did not want to supervise dental hygiene students in these procedures:

*“The dental supervisor*,* who supervises the cluster*,* transfers this task to his colleague dental supervisor because he does not want to supervise the dental hygiene students in preparation and restoration.”* (Table 5-7).

#### Challenge 8: initiative and leadership

A dental hygiene student noted that while opportunities for collaboration exist, active participation and initiative are essential. Additionally, it was also observed that dental students often take the lead and manage many tasks, requiring dental hygiene students to make focused contributions. This dynamic sometimes leads dental hygiene students to perform tasks independently, making collaboration less effective. This is also true when there is insufficient comprehensive dental knowledge to contribute. One student emphasised the need to take initiative to engage in collaboration:

*“Yes*,* well*,* I’m already finding that of course you also have to take your own initiative to want to collaborate. You do just get the opportunity to do it*,* but you have to tackle it yourself.”* (Table5-8).

#### Challenge 9: physical reach

In the clinic, there was physical distance between students from both programmes. The space was arranged so that students from the same programme sat near their supervisor. Generally, the interprofessional team members were able to find each other (Table 5-9).

#### Challenge 10: time constraints

Due to time constraints, mutual support sometimes came under pressure. Students often had full schedules with patient appointments (Table 5-10).

### Subject-Object

#### Challenge 11: already knowing each other

Collaborating and helping each other did become easier if they already knew each other and were able to approach each other in the clinic (Table 5-11a). Motivation to work together is boosted if there is a good atmosphere (Table 5-11b). One student reflected on how prior acquaintance facilitated collaboration:

*“That certainly makes it easier. Yes*,* if you already have good contact*,* you’re more likely to approach each other saying*,* I have this for you. Or*,* we already had a treatment plan together anyway. So*,* I said*,* can you help me with this*,* and then you can help with other things*,* like the required preparation/restorations*,* soon.”* (Table 5-11a).

## Discussion

This study examined collaborative behaviours and interprofessional learning within a multi-level workplace learning environment, identifying behaviours such as utilising expertise, active engagement, shared leadership, problem-solving, and guidance. These behaviours underscore the role of teamwork and leadership in effective IPE. Using the CHAT model, we analysed how various dynamics in a learning environment impact IPE. These dynamics included student uncertainty, hierarchical feedback structures, case management dynamics, programme requirements, conflicting expertise, task perspectives, supervision, leadership, physical proximity, time constraints, and peer relationships. These insights reveal both challenges and opportunities for enhancing IPE and strengthening collaborative practices in SRDC settings.

### Gaps in competencies

Analysing observed behaviours alongside interview data, through the lens of the four core competencies [9], provided insights into their application within this learning environment. We found that observed behaviours were closely aligned with the competencies of *Roles and Responsibilities* and *Communication*. Our findings align with prior research on IPE in student-led clinics [40], which highlighted benefits such as improved understanding of professional roles, enhanced teamwork, improved patient-centred care, and personal development in communication skills.

In contrast, the competencies of *Values and Ethics* and *Teams and Teamwork* were observed less frequently in the SRDC. Addressing these competencies is particularly crucial in an evolving landscape. Expected behaviours related to these competencies often require specific expertise [41, 42], including structured guidance, an understanding of professional cultures, reflective practice, and advanced understanding of teamwork [43]. Reflective practice is commonly embedded in curricula across healthcare fields; however, in oral healthcare, especially within the context of the SRDC, its development may not be as pronounced. This lack of emphasis on reflective practice could explain the observed gaps in competencies related to teamwork and values, which are essential for fostering a collaborative IPE environment. These competencies require not just technical expertise but also the ability to reflect on one’s role within a team, appreciate the perspectives of others, and engage in constructive dialogue. The identified gaps in competencies underscore the need for targeted interventions that focus on enhancing reflective practice within the SRDC. These findings also contribute to answering the second research question by illustrating what students appeared, and what they not yet have learned, through their engagement in collaborative behaviours within the SRDC. Moreover, they highlight the importance of understanding the factors that facilitate or hinder the development of collaborative behaviours, which is essential for effectively addressing challenges in IPE (RQ3).

### Influence of the learning environment

The influence of contextual factors on collaborative behaviour, central to the third question, was evident throughout our analysis. Several context dynamics influenced collaboration in interprofessional teams, including student uncertainty, strict hierarchical feedback structures, case management dynamics, and tensions arising from overlapping duties (e.g., conflicting areas of expertise, initiative, and leadership) (Fig. 1-C). For instance, dental hygiene students frequently reported feeling less knowledgeable than their dental peers, and the dominance of sixth-year students in discussions discouraged younger students from engaging, creating an unsafe learning environment that impeded student learning. Recognising these contextual dynamics, which affected the safety of the learning environment, aligns with findings from other research [10, 34, 39, 44, 45]. 

Additionally, divergent perspectives on labour division emerged, with dental students typically assuming the case manager role, despite both groups being qualified [4]. Not all students seem to fully understand the implications of current task redistribution for oral healthcare. Furthermore, supervisors in the SRDC, who are actively engaged in professional practice [46], shaped students’ perceptions of task division, which can create tensions when responsibilities overlap. These tensions, often influenced by informal norms, may restrict the consideration of alternative approaches and negatively impact the learning process. Due to time constraints, physical separation within the clinic, and the current structure of the ACR sessions, these influencing factors may not receive adequate attention. To address these challenges, structured supervision strategies that promote open discussions about role division and collaborative behaviour are essential. Supervisors can facilitate reflection and provide practical examples from their interprofessional experiences to mitigate role ambiguity [47]. This approach helps students understand the contributions of diverse professional expertise to patient care, fostering a more inclusive and effective interprofessional team [47, 48]. 

Although this study did not focus on broader healthcare outcomes, our findings align with the goals of the Quadruple Aim. The persistent separation between oral health and general healthcare hinders integrated and person-centred care. Promoting interprofessional collaboration within oral healthcare education, such as in a SRDC, contributes to bridging this gap by preparing a workforce for integrated care delivery. This aligns with the FDI Vision 2030, which calls for integration of oral health into general healthcare and for strengthening collaboration in practice [2, 27]. Nevertheless, the integration of general and oral health as envisioned in FDI Vision 2030 requires collaboration that extends beyond dentistry and dental hygiene alone.

Creating dedicated time for reflection on professional roles and teamwork can further assist students in internalising the *values and ethics* of interprofessional practice, enhancing their confidence, competence, and motivation to engage actively within the team, ultimately improving outcomes in IPE [10]. 

### Educational programme

The two educational programmes operate as distinct activity systems governed by separate written and unwritten rules, converging within the single activity system of the SRDC. This convergence often leads to tensions, especially when students from different programmes are required to perform overlapping treatments (Fig. 3-B). Such overlap can foster competition in the execution of certain tasks. While collaboration was driven by programme requirements, challenges arise from overlapping responsibilities and differing perceptions of expertise, leading to conflicts in task division. For instance, dental students underestimated the full scope of dental hygiene practice, reducing it to dental cleaning, which exacerbates tensions.

Analysis through the CHAT framework revealed differences between the two programmes that affected collaborative behaviour, contradictory to changes in laws and regulations. Our findings indicated that although students worked together within the shared SRDC framework, the differing mandates of their programmes often resulted in misaligned roles and responsibilities, creating uncertainty regarding professional boundaries. This aligns with existing literature, which identifies “role blurring”, confusion over boundaries and responsibilities as barriers to effective teamwork [49]. These blurred boundaries, along with hierarchical structures in role assignments, can significantly impact collaboration across training levels (Fig. 3-A). Research supports the notion that hierarchy influences collaborative behaviour across disciplines [12, 15, 50]. According to CHAT, contradictions—tensions within or between distinct systems—can catalyse positive change when effectively managed, but if unresolved, they may impede progress [36]. 

### Implications for practice

The SRDC offers a structured, authentic interprofessional learning environment, emphasizing small-group discussions and collaborative patient care, fostering students’ learning *with*, *from*, and *about* each other [8]. However, communication within the SRDC primarily centres on technical patient issues, with limited focus on interprofessional dialogue and reflection on collaborative practice, which are essential for understanding professional culture and differences [49]. Supervisors encounter challenges in modelling effective interprofessional collaboration in evolving professional fields [51]. They would benefit from support in enhancing facilitation skills through co-teaching, sharing interprofessional experiences, and self-assessment of IPE effectiveness[47].

### Strengths and limitations

This study offers insights into collaborative behaviours that support effective teamwork in a SRDC, where dental hygiene and dental students engage in multi-level learning. Through observations and interviews, triangulated by research team members with diverse backgrounds and using frameworks such as IPE CORE competencies and the CHAT framework, key factors and potential collaborative tensions were identified. These findings highlight opportunities to enhance the learning potential within this interprofessional environment. While the study is limited to a single SRDC in the Netherlands, it underscores how contextual dynamics influence collaborative behaviour and interprofessional learning. The first author’s dual role as teacher and researcher may have influenced the findings; however, reflexive discussions within the research team and structured observation guides helped to minimise bias and strengthen the study’s credibility. The use of the IPEC competencies and the CHAT framework provided a clear analytical structure but may also have steered the interpretation of the data. These frameworks represent one possible lens for understanding interprofessional learning, and alternative theoretical perspectives might have yielded different insights.

It is important to note that the study focuses exclusively on collaboration within an educational setting designed for interprofessional education. Factors such as age, gender, and other socio-demographic variables, which may affect collaboration in professional clinical dental practice, were beyond the scope of this study.

Based on our findings, we suggest strategies such as structured reflective practice sessions, targeted supervisor training, and facilitation of role negotiation in the teams. These approaches could be further developed or tested in other IPE oral healthcare settings to evaluate broader relevance and impact. Future research could also explore how these collaborative behaviours relate to patient outcomes.

This study focused exclusively on collaboration between dental and dental hygiene students, as these were the only professional groups working together in the clinical setting. It would be interesting to explore how to involve other healthcare professionals meaningfully in the SRDC and to study how dynamics would change.

## Conclusion

In conclusion, clear role division and shared leadership fostered a structured and respectful learning environment, while role delineation, reciprocal supervision, and active knowledge exchange supported a productive clinical atmosphere for development for interprofessional behaviours. However, more integrated and balanced strategies are needed to ensure equitable benefits for all students in collaborative learning. This study highlights how two distinct curricula in an evolving professional landscape shaped students’ collaborative behaviours within an interprofessional learning environment in which there are both written and unwritten rules. Addressing challenges like enhancing communication, promoting continuous feedback, refining supervisory roles, and encouraging role diversity may further optimise interprofessional education, ultimately benefiting patient care and team cohesion. Finally, this study underscores the importance of responsive education that aligns with the dynamic nature of healthcare practices. To meet the evolving demands of the interprofessional healthcare environment, curriculum adjustments may be necessary to prepare students for collaborative practice, ensuring they are sufficiently equipped. By incorporating these findings into practice, educational programmes can better foster collaborative skills, improve interprofessional relationships, and ultimately enhance both learning outcomes and patient care.

## Supplementary Information

Supplementary material 1: Topic list of Observations.

Supplementary Material 2: Interview guide.

Supplementary Material 3: Tables 1, 2, 3, and 4.

Supplementary Material 4: Table 5.

Supplementary Material 5: List of abbreviations.

## Data Availability

The datasets generated and/or analysed during the current study are not publicly available due to privacy or ethical restrictions but are available from the corresponding author on reasonable request.
