# Supplementary material for: Dynamics in interprofessional learning: a focussed ethnographic study in a student-run dental clinic
Source: BMC Med Educ. 2025 Dec 19;26:127. doi: 10.1186/s12909-025-08383-1 (PMC12831374; doi:10.1186/s12909-025-08383-1)
Supplement: Supplementary file 1 — Supplementary material 1: Topic list of Observations. [file 12909_2025_8383_MOESM1_ESM.docx]

**Interviewguide Student-Run Dental Clinic (SRDC)**

| **Introduction** |  |
| --- | --- |
| **Introduction** | Explanation of the interview in relation to the observation. |
| **Purpose of the study** | The purpose of this research is to gain insight into the behavior, interactions, and mechanisms in interprofessional collaboration between students of oral health care and dentistry in the SRDC learning environment, which influences interprofessional learning and collaboration. |
| **Confidentiality** | The data will be pseudonymized, stored, and kept secure. |
| **Stopping early** | You can stop the research at any time, without providing a reason. Any data collected up until that point will still be included in the study. |
| **Recording** | The interview will be recorded using audio recording equipment. The audio files will be deleted within 6 months. The transcriptions of the audio recordings will be kept for 10 years in a secure research environment. |
|  |  |

The in-depth interview consists of follow-up questions, which have been developed by the researcher in consultation with the research team. Over time, the interview guide may be refined in collaboration with the research team if necessary. This interview guide provides examples of questions that may be asked and gives an overview of how the interview will generally be structured. It also indicates the goals of the various questions.
After the introduction, the researcher describes an observed meaningful event. The researcher then asks follow-up questions to gain insight into the underlying mechanisms, the role of the context in those mechanisms, and the relationship between interpersonal interactions, interprofessional communication, and interprofessional outcomes.
Once the meaningful event has been sufficiently explored, if relevant, a subsequent meaningful event may be discussed, and these can be compared to gain deeper insights and identify patterns.

| **Description** | Goal |
| --- | --- |
| **Opening** | "I have observed you during interprofessional collaboration in a meaningful event related to patient care, such as the intake, treatment planning, treatment plan presentation, performing treatment, and evaluation. How do you look back on this?"  "How did you experience it? Were you yourself in this collaboration?" |
|  |  |
| **Core** | The researcher briefly describes the observed meaningful event. (For example: "You performed an intake with A., created a treatment plan, and presented the plan.") |
| Then, the researcher asks follow-up questions to gain insight into the underlying mechanisms and the role of the context in those mechanisms. | Obtain information about what is not observable.  And for the researcher, validate the observations made. |
| "How do you look back on it? What do you remember?" | Validate observations with the respondent's experience. |
| "What made you…? What were the reasons for you…?" | Explore considerations. |
| "What did you think about it? How did you experience…?" | Explore attitudes/feelings. |
| "What did it provide for the patient? What made that happen?" | Explore the impact on the patient. |
| "How did you experience this role? What made you take on this role?" | Explore role and personal involvement. |
| "What did it teach you? What made that happen?" | Explore learning outcomes. |
| "Has this meaningful event changed the way you think about…?" | Explore changes in perspective. |
| "Have you done anything differently as a result of this meaningful event?" | Explore behavioral changes due to the event. |
| "What do you need to achieve this?" | Explore resources needed for success. |
| **After** the meaningful event has been sufficiently explored, a subsequent event may be discussed, and events can be compared to gain deeper insight and identify patterns. |  |
| "Was this meaningful event an example of how things normally go?" | Insight into normal vs. exceptional situations. |
| If different: "What was different? What made it different than usual?" | Compare and analyze deviations. |
| "In meaningful event A, this happened, but in meaningful event B, it went like this." | Explore differences and patterns between events. |
| "What made the situation different? What made you react this way…?" | Gain insight by comparing meaningful events. |
| **Closing** | "What would you still like to add about interprofessional learning and collaboration in this learning environment?" |
| "Is there something that has not been addressed or is insufficiently covered?" | Ensure everything the student wanted to contribute is discussed. |
| "Do you have any other questions/comments?" | Check if the student has any final questions or remarks. |
