# Supplementary material for: Dynamics in interprofessional learning: a focussed ethnographic study in a student-run dental clinic
Source: BMC Med Educ. 2025 Dec 19;26:127. doi: 10.1186/s12909-025-08383-1 (PMC12831374; doi:10.1186/s12909-025-08383-1)
Supplement: Supplementary file 2 — Supplementary Material 2: Interview guide. [file 12909_2025_8383_MOESM2_ESM.docx]

**Topic List of Observations during Meaningful Events in Patient Care where Interprofessional Collaboration is Needed in the Student-Run Dental Clinic (SRDC)**

| **Study subjects** |  |
| --- | --- |
| **Persons** | Dental hygiene students participating in the SRDC. |
|  | Dental students participating in the SRDC. |
|  | Teachers from both programs in Academic Clinical Reasoning (ACR) sessions and during the clinic: They are indirect subjects of this study. No personal data is collected in this study. |
|  | Clinic (reception) assistants during the clinic: They are indirect subjects of this study. No personal data is collected in this study. |
|  | Patients from the SRDC: They are indirect subjects of this study in the event if they play a role as a patient in the interaction with the students. No patient data is collected in this study. |
| **Meaningful Events** | Intake, treatment planning, treatment plan presentation, performing treatment, and evaluation. |
| **Written Materials/Artifacts** | Email, app contact, etc. |

What behavior and interactions do dental hygiene and dental students demonstrate in collaboration during interprofessional learning and working in the SRDC?

What mechanisms underlie the behavior and interactions of dental hygiene and dental students in collaboration during interprofessional learning and working in the SRDC?

| Topic List |  |  |
| --- | --- | --- |
| Topic | Sub topic | Explanation |
| Descriptive observation |  |  |
|  | Space | What are the characteristics of the space (ACR room, clinic, or digital contact between students via email or app)? |
|  | Context | What is the meaningful event being observed?  What is the moment of interprofessional contact?  What is the reason for interprofessional contact?  What is the content of the task? |
|  | Interprofessional Collaboration | **Actors and Roles**  • Which persons are involved?  • What is the role division of the involved persons?  • What are the goals of the involved persons?  **Artifacts**  • What is being used? (tools, questionnaires, etc.) |
|  | | |
| Focused Observation |  |  |
|  | Communication (verbal and non-verbal) | What is being said?  What is not being said?  What language do the participants use (e.g., jargon)?  What non-verbal communication is observed?  What communication is observed regarding learning?  What communication is observed regarding collaboration? |
|  | Interprofessional Collaboration | **Interaction**  • Is there interaction?  • Who is involved in the interaction?  **Behavior**  • What behavior is observed?  • What behavior regarding learning is observed?  • What behavior regarding collaboration is observed?  **Emotions**  • What emotions are expressed by the participants? |
|  | | |
| Mechanisms | | |
|  | Feeling responsible | \|  \| \| --- \| \| Authentic case.  Use of discipline-specific knowledge.  Personality traits. Actively involved in interaction. \| \| |
|  | Feeling enthusiastic/excited | Task, role, and learning goals align with knowledge and skill level (motivation).  Enthusiasm about the task and collaboration with students from other disciplines. Mutual interactions. |
|  | Feeling safe | Willingness to give opinions.  Informal learning environment. |
|  | Feeling ready | Feeling confident about profession-specific knowledge (autonomy).  Trust in one's own knowledge (competence).  Contribution to the team (recognized and valued).  Lots of interaction possible. |
| Selective Observation |  |  |
|  | Contrast | Accent on difference or paradox. What is the difference? What has changed? |
|  | Discovery | What have I not seen before? |
|  | Time | How long has the observation lasted? |
|  |  |  |
| Observer reflection | Reflection | How do I experience the observation? (emotions, interpretations, interactions, problems) |
