# Supplementary material for: Dynamics in interprofessional learning: a focussed ethnographic study in a student-run dental clinic
Source: BMC Med Educ. 2025 Dec 19;26:127. doi: 10.1186/s12909-025-08383-1 (PMC12831374; doi:10.1186/s12909-025-08383-1)
Supplement: Supplementary file 3 — Supplementary Material 3: Tables 1, 2, 3, and 4. [file 12909_2025_8383_MOESM3_ESM.docx]

**List of abbreviations**

**ACR:** Academic Clinical Reasoning

**C:** Communication

**CHAT:** Cultural-Historical Activity Theory

**DHS:** Dental Hygienist students

**DS:** Dental Students

**EPAs:** Entrustable Professional Activities

**IPE:** Interprofessional education

**IPEC:** Interprofessional Education Collaborative

**RR:** Roles and Responsibilities

**RQ:** Research question

**SRDC:** Student-Run Dental Clinic

**TT:** Teams and Teamwork

**VE:** Values and Ethics

**VWS:** Volksgezondheid, Welzijn en Sport
