# Supplementary material for: Dynamics in interprofessional learning: a focussed ethnographic study in a student-run dental clinic
Source: BMC Med Educ. 2025 Dec 19;26:127. doi: 10.1186/s12909-025-08383-1 (PMC12831374; doi:10.1186/s12909-025-08383-1)
Supplement: Supplementary file 4 — Supplementary Material 4: Table 5. [file 12909_2025_8383_MOESM4_ESM.docx]

| **Table 1** | **Core Competence Values and Ethics with illustrative quotations** | |
| --- | --- | --- |
| Core Competence | Description (subcompetences) | Quotations |
| **Values and Ethics** | **Work with team members to maintain a climate of shared values, ethical conduct, and mutual respect** |  |
| VE1 | Promote the values and interests of persons and populations in health care delivery, One Health, and population health initiatives. | NA |
| VE2 | Advocate for social justice and health equity of persons and populations across the life span. | NA |
| VE3 | Uphold the dignity, privacy, identity, and autonomy of persons while maintaining confidentiality in the delivery of team-based care | NA |
| VE4 | Value diversity, identities, cultures, and differences |  |
| VE5a | Value the expertise of health professionals and its impacts on team functions and health outcomes | e.g.: I: I think our group always participates quite actively and asks a lot of questions and thinks along well. Yes, I like that. I just had some things myself that I was in doubt about and then it's nice to get a bit of input back as well of have you thought about that or would you not do it that way. (D1-1) |
| VE6. | Collaborate with honesty and integrity while striving for health equity and improvements in health outcomes |  |
| VE7a | Practice trust, empathy, respect, and compassion with persons, caregivers, health professionals, and populations | e.g.: FN: Meanwhile, the scanning equipment has been set up. The dental student now sits next to the patient. The dental hygiene student assists by suctioning during the scan. The dental student begins scanning and, in the meantime, the dental hygiene student asks the patient, “Are you still comfortable?” The dental student asked the dental hygiene student if she would like to scan the upper jaw. The dental hygiene student takes over, and the dental student gives instructions and then takes over again. The dental hygiene student says, “It looks easy, but it’s not simple. (DT22-1 and DH17-3) |
| VE8 | Apply high standards of ethical conduct and quality in contributions to team-based care | NA |
| VE9 | Maintain competence in one’s own profession in order to contribute to interprofessional care | NA |
| VE10a | Contribute to a just culture that fosters self-fulfillment, collegiality, and civility across the team | e.g.: FN: The dental student said, “Go ahead and measure the mobility as well.” The dental student mentions a tooth and says, “I think the mobility there is 1.” The dental hygiene student checks and does not find this measurement. The dental student observes how the dental hygiene student performs the measurement and says, “We need to feel the mobility between the ends of two instruments.” She demonstrates to the dental hygiene student, who acknowledges, “Oh, like this.” The dental student says, “Then I do think it is mobile.” The dental student actively observes the dental hygiene student and records the results in the computer. (D6-1 and DH14-3) |
| VE10b |  | e.g.: FN: Colloquium room (see attachment: photo with permission from the students and sketch of the room layout with attendees) (VE10). |
| VE11a | Support a workplace where differences are respected, career satisfaction is supported, and well-being is prioritized | e.g.: FN: Meanwhile, the scanning equipment has been set up. The dental student now sits next to the patient. The dental hygiene student assists by suctioning during the scan. The dental student begins scanning and, in the meantime, the dental hygiene student asks the patient, “Are you still comfortable?” The dental student asked the dental hygiene student if she would like to scan the upper jaw. The dental hygiene student takes over, and the dental student gives instructions and then takes over again. The dental hygiene student says, “It looks easy, but it’s not simple. (DT22-1 and DH17-3) |
| FN = Fieldnote  I = Interview e.g. = For example  NA = Not Applicable  DHx - 3, DHx-4 = dental hygiene student, x=participant number, the number – 3, - 4 = year level bachelor  Dx-1, Dx-2, Dx-3 = dental student, x = participant number, -1,-2,-3 = year level master | | |

| **Table 2** | **Core Competence Roles and Responsibilities with illustrative quotations** | |
| --- | --- | --- |
| Core Competence | Description (subcompetences) | Qutotations |
| **Roles and Responsibilities** | **Use the knowledge of one’s own role and team members’ expertise to address individual and population health outcomes** |  |
| RR1a | Include the full scope of knowledge, skills, and attitudes of team members to provide care that is person-centered, safe, cost-effective, timely, efficient, effective, and equitable | e.g.: I: We collected the data together. We gather the treatment plan data beforehand. She had taken the light photos, made the scan, and taken the X-rays, which were smaller tasks. I completed the periodontal status myself, but she was present and helped with that. She also heard the advice and instructions I provided during that session, and vice versa. (DH7-4) |
| RR1b |  | e.g.: FN: The D supervisor then shares his perspective on the technical problem with the patient. A discussion ensues within the group, and the DH student (presenter) responds to this. (D4-2 and DH4-3) |
| RR1c |  | e.g.: FN: The dental student starts scanning the mouth while the dental hygiene student observes. The dental student sits on the mobile operator's chair, and the dental hygiene student stands next to the chair where the patient is seated. During the scan, the dental hygiene student assists with the procedure. (D19-1 and DH17-4) |
| RR1d |  | e.g.: FN: A while later, another patient comes in for treatment with the dental hygiene student, in the same combination with the dental student. There is a very brief communication when the patient and the dental student come to the dental hygiene student's unit. The patient sits down, and the dental student then says audibly to everyone present in the unit (patient, dental hygiene student, and observer), "It looked neat. Next time we'll take a bitewing." The dental student walks away and says to the patient, "Good luck, and I'll see you next time. (D20-2 and DH18-4) |
| RR1e |  | e.g.: FN: Based on a paper plan, the dental student begins explaining the treatment plan. He explains how the treatment plan is structured and makes regular eye contact with the patient. He tells the patient that the main issue for him is the caries: "For you, the biggest problem is tooth decay." The patient nods and listens. Then the dental student asks the dental hygiene student: "You can explain this better." The dental student explains the periodontal status and mentions that she has performed various measurements, including those related to bleeding and plaque scores. The patient follows along with the paper plan that is being referred to (D21-2 and DH19-4) |
| RR1f |  | e.g.: FN: The dental hygiene student gives instructions to ensure better access in the mouth. The dental student said: "Turn a bit to the right?" The treatment is then continued with hand instruments. The dental student asks: "Which instrument is best to use?" Different options are discussed. Then, they start. The dental hygiene student: "Is there a reason why you're starting there?" The dental student: "No." Midway, they switch positions at the chair. The dental student: "Would you do it differently? Or would you do it this way too?" The dental hygiene student stands next to her and gives tips. The dental student to the patient: "Are you still okay?" Patient: "Yes." The dental student asks the dental hygiene student to check inside the mouth. Both students switch positions. The dental hygiene student inspects. The dental student shrugs and nods her head. The dental hygiene student indicates that it still feels rough and that there could be several causes. "It could be a rough root surface... but I'm not getting anything else out." The suggestion is to ask the instructor for assistance. (D22-1 and DH9-4) |
| RR1g |  | e.g.: FN: The dental hygiene student starts the treatment. "How would you begin?" she asks the dental student. The dental student demonstrates in the mouth how she would place the drill. The dental hygiene student then asks, "Would you start here?" After consulting with the dental student, the dental hygiene student begins the treatment. The dental student handles suctioning and observes the procedure. She follows all the actions closely. Midway, the dental student observes and occasionally sits in the dental hygiene student's position for a better view. As the treatment progresses, the dental student fetches the instructor (she adds her name to the list the instructor carries, indicating when guidance is needed) to check if the enamel is clean. The dental supervisor has observed and the dental hygiene student can continue with the treatment. During the subsequent steps, the dental hygiene student actively asks the dental student questions on how to proceed with the treatment. For instance, when placing the band for the restoration, the dental student demonstrates and holds it in place. The dental hygiene student stands by, helps, and observes the mouth. The dental student then provides instructions during the finishing process and assists the dental hygiene student, who frequently seeks advice. Meanwhile, when removing the cotton rolls, the dental hygiene student says to the patient, "You’re having a hard time, aren’t you?" The patient stretches and responds warmly to the dental hygiene student. The dental hygiene student moves on to the next tooth and asks the dental student, "Would you start here?" The dental student swaps chairs with the dental hygiene student for a better view. The dental hygiene student asks, "It needs to be a bit larger, doesn’t it? Is it completely clean now?" The dental student points out a spot, and the dental hygiene student says, "I'll just remove that speck," and continues the preparation. I stopped the observation after that. I was able to ask the dental hygiene student a few brief questions. The dental hygiene student mentioned that she found it helpful when the dental student asked, "What do you think?" The dental hygiene student said, "It makes me think more. (D7-4 and DH17-3) |
| RR1h |  | e.g.: I: Very much so actually, because yes, she gave good tips. They obviously do those treatments much more often and we do them a bit less often and do more complex treatments so yes, if you do that again like that now and she has some useful tips on how best to put the patient down, or yes, I don't know. That did help me a lot in cleaning properly’. (D11-1) |
| RR1i |  | e.g.: I: *What is the learning effect for you, for example in this particular event?* ' You become aware of what you yourself normally do somewhat automatically anyway. So you do learn to think about the steps again. And when they ask something, you naturally have to think: well, why do you do it like that? Or I think that's mainly the effect it has. Because normally, of course, you are working by yourself with an assistant next to you who follows, well, and in this way you are also coaching a little and watching and giving some tips.(D24-3) |
| RR2a | Collaborate with others within and outside of the health system to improve health outcomes. | e.g.: I: I think our group always participates quite actively and asks a lot of questions and thinks along well. Yes, I like that. I just had some things myself that I was in doubt about and then it's nice to get a bit of input back as well of have you thought about that or would you not do it that way. (D1-1) |
| RR3a | Incorporate complementary expertise to meet health needs including the determinants of health | e.g.: I: Well, it just provides a lot of knowledge, because then you can also work interprofessionally and then you can also look at and think of different problems, which so say yourself cannot see, from different angles on a daily basis, and also say learn. (DH8-4) |
| RR4a | Differentiate each team member’s role, scope of practice, and responsibility in promoting health outcomes | e.g.: I: But what I do always actually try to do is to present together with a dental hygiene student, because actually there is always something of education, instruction, behavioural change that always come with it in one way or another. So I do think it's important that oral health is involved. (D8-2) |
| RR4b |  | e.g.: FN: The next part of the measurement is staining the dental plaque, and the dental hygiene student explains this to the patient. While the measurements are being taken, the dental student enters the data into the computer. During the furcation measurement that the dental hygiene student is performing, the dental student asks, “Are you also checking the bitewing (X-ray)?” She shows it on the computer screen to the dental hygiene student. They discuss it briefly together, and then it is recorded. Interpretation: Active exchange of knowledge. (D19-1 and DH17-4) |
| RR5. | Practice cultural humility in interprofessional teamwork. | NA |
| FN = Fieldnote  I = Interview  e.g. = For example  NA = Not applicable  DHx - 3, DHx-4 = dental hygiene student, x=participant number, the number – 3, - 4 = year level bachelor  Dx-1, Dx-2, Dx-3 = dental student, x = participant number, -1,-2,-3 = year level master | | |

| **Table 3** | **Core Competence Communication with illustrative quotations** | |
| --- | --- | --- |
| Core Competence | Description (subcompetences) | Quotations |
| **Communication** | **Communicate in a responsive, responsible, respectful, and compassionate manner with team members.** |  |
| C1a | Communicate one’s roles and responsibilities clearly | e.g.: FN: It seems that the presenters primarily present within their own areas of expertise. (D9-2 and DH16-3) |
| C1b |  | e.g.: FN: The D student takes on a large part of the presentation. The DH student observes and looks at the D student with a neutral expression. (D9-2 and DH16-3) |
| C1c |  | e.g.: I: We collected the data, together and sat down together of okay, what did we all see? And we wrote it together and just put the presentation together as well. Don't say one person did almost nothing and another worked a lot. We just picked up everything together and that was really nice.(DH14-3) |
| C1d |  | e.g.: FN: A while later, another patient comes in for treatment with the dental hygiene student, in the same combination with the dental student. There is a very brief communication when the patient and the dental student come to the dental hygiene student's unit. The patient sits down, and the dental student then says audibly to everyone present in the unit (patient, dental hygiene student, and observer), "It looked neat. Next time we'll take a bitewing." The dental student walks away and says to the patient, "Good luck, and I'll see you next time. (D20-2 and DH18-4) |
| C1e |  | e.g.: I: The patient had expressed a preference for a constant practitioner in the treatment. That is why the dental student is doing the periodic oral examination. The dental student has been attached to the clinic for more years than the dental hygiene student and is the case manager of the patient ". (DH18-4) |
| C1f |  | e.g.: I: I always try to observe because a handover alone is insufficient. This way, I often have already a better understanding of what is going on in the patient’s mouth (DH3-3) |
| C1g |  | e.g.: FN: Based on a paper plan, the dental student begins explaining the treatment plan. He explains how the treatment plan is structured and makes regular eye contact with the patient. He tells the patient that the main issue for him is the caries: "For you, the biggest problem is tooth decay." The patient nods and listens. Then the dental student asks the dental hygiene student: "You can explain this better." The dental student explains the periodontal status and mentions that she has performed various measurements, including those related to bleeding and plaque scores. The patient follows along with the paper plan that is being referred to (D21-2 and DH19-4) |
| C2 | Use communication tools, techniques, and technologies to enhance team function, well-being, and health outcomes. | NA |
| C3a | Communicate clearly with authenticity and cultural humility, avoiding discipline-specific terminology. | e.g.: FN: An extensive technical discussion arises about the fabrication of the frame. The D students in the room, the D student (who also contributed to the presentation), and the D supervisor engage in an in-depth discussion. The DH students do not participate in this. The DH supervisor joins the conversation and also asks questions. (D3-3 and DH3-3) |
| C4a | Promote common understanding of shared goals. | e.g.: I: We collected the data, together and sat down together of okay, what did we all see? And we wrote it together and just put the presentation together as well. Don't say one person did almost nothing and another worked a lot. We just picked up everything together and that was really nice. (DH14-3) |
| C5a | Practice active listening that encourages ideas and opinions of other team members | e.g.: FN: The presentation starts at 8:54. The DH student opens the presentation. Then, the D student takes over part of the presentation again, looking at the projection screen. As the DH student continues, she talks about the patient, whom they suspect might be using cannabis. She says to the D student: “Maybe you can explain that better?” They laugh at each other. Overall, the students are presenting their story to the group with smiles. The group listens attentively, is silent, and looks at the presentation from their fellow students. (D7-1 and DH7-4). |
| C5b |  | e.g.: FN: The discussion in the group then turns to where the treatment should start given the circumstances (financially) and the patient's wishes. Overall, the group comes to the consensus that a healthy oral situation should be prioritized, even if the patient desires better aesthetics. The DH student (presenter) states that she agrees with this in the discussion. The D student then summarizes what the plan will be. (D4-2 and DH4-3 |
| C5c |  | e.g.: I: I think our group always participates quite actively and asks a lot of questions and thinks along well. Yes, I like that. I just had some things myself that I was in doubt about and then it's nice to get a bit of input back as well of have you thought about that or would you not do it that way. (D1-1). |
| C6a | Use constructive feedback to connect, align, and accomplish team goals. | e.g.: I: I also actually try to always present all the plans so that I at least and get feedback from the lecturers and get feedback from the students that it's also a bit of a mirror of okay, what does everyone else think about it?.... I do think it's important. Let's say what the group thinks of that. (D8-2) |
| C6b |  | e.g.: FN: The D supervisor (male) agrees with this. He then asks the group: “I understand that we need to look at the oral level, but are there any learning moments at the tooth level?”(D9-2 and DH16-3) |
| C6c |  | e.g.: I: I also actually try to always present all the plans so that I at least and get feedback from the lecturers and get feedback from the students that it's also a bit of a mirror of okay, what does everyone else think about it?.... I do think it's important. Let's say what the group thinks of that. (D8-2) |
| C6d |  | e.g.: I: (interviewer) But I was just curious about how you did that preparation. Did you and this student see the patient together? How did that go? Can you explain that?  Student: Regarding this treatment plan presentation, I wasn't present with this patient, so I only worked on it by sharing knowledge and discussing the patient's problems.  Still, the collaboration in this presentation was a bit difficult and I clearly discussed that with the dental student. He had very little time. ...... So in that respect I did give a bit of criticism towards my fellow student he also thought it was justified. *Interviewer*: What was your criticism. Yes, that indeed so we couldn't work together so super often and that say sometimes the points became difficult to exchange. But in the end, I think the final product was good. So well, the collaboration was not so super easy, but end point was nicely worthwhile. (DH8-4) |
| C6e |  | e.g.: I: But maybe, yes, look, if you really wanted to do it formally according to the guidelines, because DH8 hasn't seen him yet, that would actually be the intention. So ideally, we would have had him come back for another appointment with DH8. Then DH8 could also have, for instance, seen how his oral hygiene would improve if we had given him instructions and how he would follow them. However, the treatment plan would then be considerably delayed because DH8 has no availability in the coming time, and neither do I. (D8-2) |
| C6f |  | e.g.: FN: The dental hygiene student gives instructions to ensure better access in the mouth. The dental student said: "Turn a bit to the right?" The treatment is then continued with hand instruments. The dental student asks: "Which instrument is best to use?" Different options are discussed. Then, they start. The dental hygiene student: "Is there a reason why you're starting there?" The dental student: "No." Midway, they switch positions at the chair. The dental student: "Would you do it differently? Or would you do it this way too?" The dental hygiene student stands next to her and gives tips. The dental student to the patient: "Are you still okay?" Patient: "Yes." The dental student asks the dental hygiene student to check inside the mouth. Both students switch positions. The dental hygiene student inspects. The dental student shrugs and nods her head. The dental hygiene student indicates that it still feels rough and that there could be several causes. "It could be a rough root surface... but I'm not getting anything else out." The suggestion is to ask the instructor for assistance.(D22-1 and DH9-4) |
| C6g |  | e.g.: FN: The dental hygiene student starts the treatment. "How would you begin?" she asks the dental student. The dental student demonstrates in the mouth how she would place the drill. The dental hygiene student then asks, "Would you start here?" After consulting with the dental student, the dental hygiene student begins the treatment. The dental student handles suctioning and observes the procedure. She follows all the actions closely. Midway, the dental student observes and occasionally sits in the dental hygiene student's position for a better view. As the treatment progresses, the dental student fetches the instructor (she adds her name to the list the instructor carries, indicating when guidance is needed) to check if the enamel is clean. The dental supervisor has observed and the dental hygiene student can continue with the treatment. During the subsequent steps, the dental hygiene student actively asks the dental student questions on how to proceed with the treatment. For instance, when placing the band for the restoration, the dental student demonstrates and holds it in place. The dental hygiene student stands by, helps, and observes the mouth. The dental student then provides instructions during the finishing process and assists the dental hygiene student, who frequently seeks advice. Meanwhile, when removing the cotton rolls, the dental hygiene student says to the patient, "You’re having a hard time, aren’t you?" The patient stretches and responds warmly to the dental hygiene student. The dental hygiene student moves on to the next tooth and asks the dental student, "Would you start here?" The dental student swaps chairs with the dental hygiene student for a better view.The dental hygiene student asks, "It needs to be a bit larger, doesn’t it? Is it completely clean now?" The dental student points out a spot, and the dental hygiene student says, "I'll just remove that speck," and continues the preparation. I stopped the observation after that. I was able to ask the dental hygiene student a few brief questions. The dental hygiene student mentioned that she found it helpful when the dental student asked, "What do you think?" The dental hygiene student said, "It makes me think more. (D7-4 and DH17-3) |
| C7 | Examine one’s position, power, role, unique experience, expertise, and culture towards improving communication and managing conflicts. | NA |
| FN = Fieldnote  I = Interview  e.g. = For example  NA = Not applicable  DHx - 3, DHx-4 = dental hygiene student, x=participant number, the number – 3, - 4 = year level bachelor  Dx-1, Dx-2, Dx-3 = dental student, x = participant number, -1,-2,-3 = year level master | | |

| **Table 4** | **Core competence Teams and Teamwork with illustrative quotations** | |
| --- | --- | --- |
| Core Competence | Description (subcompetences) | Quotations |
| **Teams and Teamwork** | **Apply values and principles of the science of teamwork to adapt one's own role in a variety of team settings** |  |
| TT1 | Describe evidence-informed processes of team development and team practices | NA |
| TT2 | Appreciate team members’ diverse experiences, expertise, cultures, positions, power, and roles towards improving team function | NA |
| TT3a | Practice team reasoning, problem-solving, and decision-making | e.g.: I: I think our group always participates quite actively and asks a lot of questions and thinks along well. Yes, I like that. I just had some things myself that I was in doubt about and then it's nice to get a bit of input back as well of have you thought about that or would you not do it that way. (D1-1) |
| TT4a | Use shared leadership practices to support team effectiveness | e.g.: FN: The students who gave the presentation are looking at their group members who are speaking alternately. The D supervisor actively participates in the conversation with the student. The dialogue takes place within the group, and the students speak while looking towards the supervisor. (D7- 1 and DH7-4) |
| TT4b |  | e.g.: FN: The D student (presenter) now responds and agrees that it is more nuanced. The DH student nods in agreement and agrees with her co-presenter's response. Then a question is asked about the application of a cold test on the patient. The D student explains what she did to the person who asked the question. The DH student nods in confirmation after her colleague's answer. (D4-2 and DH4-3) |
| TT4c |  | e.g.: I: He had initially come up with different solutions for this patient, more towards surgery and things like that. So based on that, we were just able to spar well that yes, that one or the other conceived path is not really appropriate for this patient. So, I do feel that I also had direction in writing this treatment plan. (DH8-4) |
| TT5 | Apply interprofessional conflict management methods, including identifying conflict cause and addressing divergent perspectives | NA |
| TT6a | Reflect on self and team performance to inform and improve team effectiveness | e.g.: I: Yes, by working together we end up having a better treatment plan than if you were to do it separately. That you still have two views. Of dentistry and oral health together also in areas of dentistry, dental hygiene can assist and vice versa that's not necessarily that we separate I, only look at the statuses and they only look at the crowns. But that also varies depending on who you work with. We discussed everything together. (DH21-4) |
| TT7a | Share team accountability for outcomes. | e.g.: FN: The dental hygiene supervisor asks a question about the pockets, which do not seem to be present but should be given the situation. The D student is somewhat surprised and says it is strange, as it was measured. ( D8-2 and DH8-4). |
| TT8 | Facilitate team coordination to achieve safe, effective care and health outcomes | NA |
| TT9 | Operate from a shared framework that supports resiliency, well-being, safety, and efficacy | NA |
| TT10 | Discuss organizational structures, policies, practices, resources, access to information, and timing issues that impact the effectiveness of the team | NA |
| FN = Fieldnote  I = Interview  e.g. = For example  NA = Not applicable  DHx - 3, DHx-4 = dental hygiene student, x=participant number, the number – 3, - 4 = year level bachelor  Dx-1, Dx-2, Dx-3 = dental student, x = participant number, -1,-2,-3 = year level master | | |
