# Supplementary material for: Dynamics in interprofessional learning: a focussed ethnographic study in a student-run dental clinic
Source: BMC Med Educ. 2025 Dec 19;26:127. doi: 10.1186/s12909-025-08383-1 (PMC12831374; doi:10.1186/s12909-025-08383-1)
Supplement: Supplementary file 5 — Supplementary Material 5: List of abbreviations. [file 12909_2025_8383_MOESM5_ESM.docx]

| **Table 5:** | **Challenges identified in collaboration during ACR Sessions** |  |
| --- | --- | --- |
|  | **Challenge** | **Example from field notes (FN) and Interviews (I) related to the challenge** |
| **A** | **Subject-Rules-Community** | |
| **1** | **Uncertainty Among Students** | e.g.: I:I notice that in the third year I did struggle a bit more with that, also because I thought maybe I had a comment of oh, yeah, that's irrelevant anyway... .(DH7-4)  Then it occurred to me that somehow another X-ray had to be taken of that tooth. And I hadn't mentioned that. But eventually that comment came through another student. So little things like that, and now I do dare to say it, I feel confident enough because of that (DH7-4) |
| **2a** | **Strict Hierarchy in Feedback** | e.g.: I: Anyway, when I came into the SRDC in my fourth year, then we had some sixth-years who pretty much said: this is wrong, so then, I thought, that's how it came across to me, at least, I don't know if that's probably how they meant it, but that's how it came across to me. So I never dared to ask so many questions .....(D25-3) |
| **2b** |  | e.g.: I: One dental student is more open to feedback, and another feels, I think, maybe more like they literally stand above you. It varies from student to student. But I had an experience with dental students, for example, with tooth preparation. What I liked about one dental student was that he let me think for myself—if you think it’s fine this way, then it’s good. He gave me the freedom to make my own choices, but still ensured it was correct, not insisting that it had to be done a specific way because otherwise, it would be wrong. Such collaboration is nice because it’s correct but in your own way, whereas some others seem to insist that things must be done a certain way, and if it’s not, then it’s wrong.(DH20-3) |
| **B** | **Subject-Rules-Community-Division of Labour** | |
| **3** | **Case management dynamics** | e.g.: I: That's dentistry always. The dental student is always the case manager and oral hygiene comes in as it were. (DH7-4) |
|  |  | e.g.: I: Yes, dentistry is actually always the case manager. I think it makes sense. As dentistry , you also do the PMOs so then you also have a bit of an overview of the patient, of the problems. So that's why you also take charge of the treatments. (D4-2)  e.g.: I:You were just though case manager. You did take the lead of that it all works out in the end. (D7-1) |
| **4** | **Program Requirements** | e.g.: I: Because I still needed those procedures, and she said, 'Oh, I have an extra patient now. We can do them together then(D11-1) |
|  |  | e.g.: I: Yes, I do notice that people think of each other and that if someone, for example, has restorations, it is checked whether they still need them for their internship or if we can easily come over. If someone needs to take photos quickly and someone else needs them, someone quickly says: 'Hey, do you have something to do? Can you help out for a moment?' and vice versa'. (DH13-4) |
| **C** | **Subject-Community-Object-Division of labour** | |
| **5** | **Conflicting Expertise** | e.g.: I: And I also do see the patients sometimes for check-ups, but that's not my main, treatment say so they are more likely to be able to draw out the patients' problems and want to write a plan on that. So they have that more often than we do. So that's why dentistry says of oh, I want to write a plan with that patient, because I've seen this and this. Do you want to help with that? (DH21-4) |
|  |  | e.g.: I: Well, if you just look purely at how much did one put on paper? How much has the other put on paper? Then it's 20, 80 or so, I guess, which makes sense, because on the one hand, there are a lot more dental problems and they can't solve that. But so what I say, the basic things like just putting in the X-rays and so on, that all takes quite a lot of work. And that well, in this case was from my side and that is usually also from the side of the dental student when I hear around me how that goes. But we also see that as a bit of a norm, I think by now, there you go, yes, you know when you start on a plan, you write the plan and then oral health science writes the piece of oral health science. (D25-3) |
|  |  | e.g.: I: Yes, I do the same thing every time and that’s basically just that the dental hygiene student does the dental hygiene part and then I finish the rest of the plan that’s basically how it always goes. (D1-1) |
| **6** | **View on tasks** | e.g.: I:We do the other things, but they really handle the cleaning and maintenance. And then patients always come for follow-up, check-ups and cleanings at dental hygiene, and for the semi-annual check-ups they come to us. Interviewer: So that is not also done by dental hygiene? The D student said: The semi-annual check-up, very rarely, they might do it once, but the next time it has to be a dental student again, but sometimes they do take over. …but difficult patients with prosthetics and things like that and frames are not handled by them for check-ups, so just the easier patients. (D11-4) |
| **7** | **Supervisory role** | e.g.: I: The dental hygiene supervisor then said to me (the observer): 'I am not a ‘registered dental hygienist’ myself, but at the request of my colleague the dental supervisor, I am assisting with administering anesthesia. The dental supervisor will sign off on this procedure in the chart'. (D11-1 and DH-3)  e.g.: FN: The dental supervisor, who supervises the cluster, transfers this task to his colleague dental supervisor because he does not want to supervise the dental hygiene students in preparation and restoration.  (D24-3 and DH6-3) |
| **8** | **Initiative and Leadership** | e.g.: FN: The dental supervisor, who supervises the cluster, transfers this task to his colleague dental supervisor because he does not want to supervise the dental hygiene students in preparation and restoration.  (D24-3 and DH6-3) |
|  |  | e.g.: I: Yes, well, I'm already finding that of course you also have to take your own initiative to want to collaborate. You do just get the opportunity to do it, but you have to tackle it yourself.. .(DH21-4) |
|  |  | e.g.: I: Yes, then I did have more input, because sometimes dental students themselves are very much of the initiative and pick up a lot themselves. And then for me as a dental hygiene student. Just look at the oral health part. And look at the overall picture what do you think about that? With a plan, where the collaboration then maybe runs a little less, yes, then you pick up a lot of things yourself, while I don't have all the knowledge of the dentistry stuff. Interviewer: No and because then how do I picture that now? When you say that from then, Look at the oral health part for a moment. Is that already written then? DH: No, make the oral health part? Put that in. What do you think about that? (DH9-4) |
| **9** | **Physical reach** | e.g.: FN: The clinic space is divided into compartments, with dental hygiene and dental students each having their own units in separate sections of the room. This layout also makes the supervising instructors for dental hygiene and dentistry visible. They have an office in the clinic area linked to this layout, and thus close to the supervising group. (D23-1 and DH4-3) |
| **10** | **Time Constraints** | e.g.: I: They have often asked if I could coach a dental hygiene student when they needed to do a preparation and restoration of a primary cavity. But so far, because my schedule is quite full, I haven't really had the time for it." (D22-4) |
| **D** | **Subject-Object** | |
| **11a** | **Already knowing each other** | e.g.: I: You knew each other before you started working together here. Okay, and does that make it easier? Yes. DH: 'That certainly makes it easier. Yes, if you already have good contact, you're more likely to approach each other saying, I have this for you. Or, we already had a treatment plan together anyway. So I said, can you help me with this, and then you can help with other things, like the required preparation/restorations, soon'.(DH11-3 ) |
| **11b** |  | e.g.: I: If you click a bit less with someone, it is then occasionally a bit more difficult to then make that collaboration go well. Then it is often slower to really sit down together, because you don't really know each other that well".(DH14-3) |
| FN = Fieldnote  I = Interview  e.g. = For example  DHx - 3, DHx-4 = dental hygiene student, x=participant number, the number – 3, - 4 = year level bachelor  Dx-1, Dx-2, Dx-3 = dental student, x = participant number, -1,-2,-3 = year level master | | |
